# Glycine decarboxylase induces autophagy and is downregulated by miRNA-30d-5p in hepatocellular carcinoma

**DOI:** 10.1038/s41419-019-1446-z

**Published:** 2019-02-25

**Authors:** Hao Zhuang, Fei Wu, Wen Wei, Yamei Dang, Baicai Yang, Xuda Ma, Feng Han, Yongmei Li

**Affiliations:** 1 Department of Hepatic Biliary Pancreatic Surgery, Cancer Hospital Affiliated to Zhengzhou University, Zhengzhou, 450000 Henan Province China; 2 Department of Pathogen Biology, School of Basic Medical Sciences, Tianjin Medical University, 300070 Tianjin, China; 3 School of Life Sciences, Chongqing University, 400044 Chongqing, China; 4 Department of Gynaecology and Obstetrics, Jiaxing Maternity and Child Health Care Hospital, Jiaxing, Zhejiang Province China

## Abstract

Glycine decarboxylase (GLDC) belongs to the glycine cleavage system and is involved in one-carbon metabolism. We previously reported that GLDC downregulation enhances hepatocellular carcinoma (HCC) progression and intrahepatic metastasis through decreasing ROS-mediated ubiquitination of cofilin. The role of autophagy in cancer metastasis is still controversial. Redox-dependent autophagy largely relies on the magnitude and the rate of ROS generation. Thus, we aimed to explore the role of GLDC in cellular autophagy during HCC progression. We showed that a high GLDC expression level is associated with better overall survival and is an independent factor for the favorable prognosis of HCC patients. GLDC overexpression significantly induced cell autophagy, whereas GLDC downregulation reduced cell autophagy. Of note, GLDC is the post-transcriptional target of miR-30d-5p. GLDC overexpression could rescue miR-30d-5p-mediated cell metastasis and increase autophagy. Furthermore, upregulation of GLDC could significantly decrease p62 expression and impair intrahepatic metastasis in vivo. Taken together, our results suggest that GLDC may play an important role to increasing miR-30d-5p-reduced autophagy to suppress HCC progress.

## Introduction

Hepatocellular carcinoma (HCC) is the sixth most common cancer globally and has a high mortality rate^1,2^. Cancer metastasis is still the main reason for the low survival rate of HCC patients^3,4^. Autophagy is an evolutionarily conserved lysosome-mediated process for the quality control of intracellular proteins, lipids, and organelles^5^. The role of autophagy in cancer metastasis is still controversial^6^. There are reports that autophagy promotes tumor progress^7–9^. Autophagy was initially considered to be a tumor suppressor and helpful for the elimination of oncogenic proteins and damaged organelles^5^. Later studies suggested that defects in autophagy were associated with a malignant phenotype in human cancers. Autophagy could be stimulated by the activation of Toll-like receptor (TLR)-dependent signaling, and synergized with TLR stimulation of antitumor immunity to control metastasis^10^. A recent study showed that an autophagy defect enhanced epithelial-to-mesenchymal transition, and metastasis transformation in gastric cancer cells^11^. The malignant phenotype of HCC has also been found to be correlated with inactivation of autophagy^12^. However, the detailed mechanisms by which autophagy affects tumor progression in HCC need further elucidation. Reactive oxygen species (ROS) could play a role as signaling molecules that activate autophagy directly and indirectly^13–15^. For example, ROS induces non-canonical autophagy by activating the extracellular regulated kinase (ERK) and c-Jun N-terminal kinase (JNK) pathways^16^. To a large extent, redox-dependent autophagy relies on the magnitude and the rate of ROS generation. In turn, ROS may be reduced by autophagy through several pathways such as the p62 delivery pathway, mitophagy pathway, and chaperone-mediated autophagy pathway^15,17–19^. Notably, our previous studies have found that glycine decarboxylase (GLDC) upregulation inhibits the production of ROS and increases the ratio of glutathione/oxidized glutathione (GSH/GSSG). The decreased GSH/GSSG ratio could be rescued by *N*-acetyl-l-cysteine (NAC) treatment in GLDC knockdown HCC cells^20^. Therefore, the function of GLDC in ROS regulation gives rise to a hypothesis that GLDC also regulates HCC invasion and metastasis via autophagy

Extensive. studies have demonstrated that GLDC is crucial for the photosynthetic efficiency in plants^21^. However, in eukaryotes, GLDC is the first step of the glycine cleavage system (GCS). Glycine is one of the major inputs for one-carbon metabolism, and excess glycine is converted into aminoacetone and methylglyoxal, which impair cell growth^22^. GLDC catalyzes the decarboxylation of glycine to yield an intermediate that is intercepted by tetrahydrofolate (THF) to liberate ammonia and generate 5,10-methylene-THF (CH_2_-THF), which drives de novo nucleotide biosynthesis and cellular methylation reactions during cell proliferation^23,24^. Glycine consumption is a feature specific to transformed cells with rapid proliferation and is assessed through metabolite analysis of the culture media, for example, across the NCI-60 cancer cell lines^25^. Until now, there have limited tumor studies on GLDC and the role of GLDC in tumorigenesis is under debate. Studies have shown that GLDC is upregulated in lung, brain, and prostate cancers. For example, GLDC drives tumor-initiating cells and tumorigenesis in non-small cell lung cancer (NSCLC) by upregulation of pyrimidine biosynthesis^26^. GLDC is highly expressed and is necessary for the proliferation in human glioblastoma multiforme^22^. Suppression of GLDC expression has the antitumor effect in PC-3 prostate cancer cell lines^27^. Recently, *GLDC* has been suggested to be a putative tumor-suppressor gene in gastric cancer^28^. Our previous study showed that GLDC upregulation increased cofilin ubiquitination and inhibited migration and invasiveness of HCC cells^20^. Therefore, it will be useful to further understand the regulation mechanisms of GLDC in HCC progress.

In this study, we demonstrated that GLDC upregulation is an independent factor for favorable prognosis of HCC patients and that GLDC enhances cell autophagy, resulting in inhibition of cell migration and invasiveness in HCC cells. In addition, we also found that GLDC is the post-transcriptional target of miR-30d-5p in HCC.

## Materials and methods

### Patients and clinical samples

Paired fresh HCC tissues and para-tumor tissues (25 pairs) were collected between January and March 2016 from the Henan Cancer Hospital Affiliated to Zhengzhou University (Zhengzhou, China)^20^. Tumor and para-tumor tissues from 94 HCC patients were collected between 2011 and 2012 from Henan Cancer Hospital Affiliated to Zhengzhou University (Zhengzhou, Henan, China). The tissues were embedded in paraffin and used for the construction of a tissue microarray. The HCC diagnosis was confirmed by pathology. Patients who died of non-liver diseases or accidents were excluded from the study. Clinicopathological characteristics of the patients are listed in Table 1. Tumor staging was defined based on the tumor node metastasis (TNM) classification system (version 4.2017) by the National Comprehensive Cancer Network (NCCN) and Barcelona Clinic Liver Cancer (BCLC) staging system. The study was conducted with the informed consent of the patients and ethics approval from the Ethics Committee (no. 2016CT054) of Henan Cancer Hospital.

**Table 1:** Clinicopathological information of 94 HCC patients

Variable	Total case	GLDC expression (%)		*P-*value
		Low	High	
All case	94	42	52	
Age				
<60	60	28 (46.7)	32 (53.3)	0.607
≥60	34	14 (41.2)	20 (58.8)	
Gender				
Male	79	35 (44.3)	44 (55.7)	0.866
Female	15	7 (46.7)	8 (55.3)	
AFP (ng/mL)				
<400	47	21 (44.7)	26 (55.3)	1.000
≥400	47	21 (44.7)	26 (55.3)	
Hepatitis				
None	8	5 (62.5)	3 (37.5)	0.265
HBV	84	37 (44.0)	47 (56.0)	
HCV	2	0 (0.0)	2 (100.0)	
Intraoperative hemorrhage (mL)				
<300	42	15 (38.1)	26 (61.9)	0.248
≥300	52	26 (50.0)	26 (50.0)	
Child–Pugh				
A	84	36 (42.9)	48 (57.1)	0.303
B	10	6 (60.0)	4 (40.0)	
Surgery time (h)				
<120	45	17 (37.8)	28 (62.2)	0.197
≥120	49	25 (51.0)	24 (49.0)	
Intraoperative blood transfusion				
No	52	20 (38.5)	32 (61.5)	0.177
Yes	42	22 (52.5)	20 (47.6)	
Tumor number				
Single	84	39 (46.4)	45 (53.6)	0.323
Multiple	10	3 (30.0)	7 (70.0)	
Tumor size (cm)				
<5	31	10 (32.3)	21 (67.7)	0.089
≥5	63	32 (50.8)	31 (49.2)	
Tumor shape				
Nodular	74	27 (36.5)	47 (63.5)	0.002^*^
Massive	20	15 (75.0)	5 (25.0)	
Capsule				
No	16	6 (37.5)	10 (62.5)	0.526
Yes	78	36 (46.2)	42 (53.8)	
Microvascular invasion				
No	43	15 (34.9)	28 (65.1)	0.079
Yes	51	27 (52.9)	24 (47.1)	
Macrovascular invasion				
No	77	32 (41.6)	45 (58.4)	0.195
Yes	17	10 (58.8)	7 (41.2)	
Satellite metastasis				
No	78	35 (44.9)	43 (55.1)	0.579
Yes	16	7 (43.8)	9 (56.3)	
Lymph nodes metastasis				
No	89	37 (41.6)	52 (58.4)	0.011^*^
Yes	5	5(100.0)	0 (0.0)	
BCLC stage				
A	50	23 (45.0)	27 (54.0)	0.023^*^
B	22	5 (22.7)	17 (77.3)	
C	22	14 (63.6)	8 (36.4)	
TNM stage				
I	49	17 (34.7)	32 (65.3)	0.042^*^
II + III + IV	45	25 (55.6)	20 (44.4)	

*AFP* alpha fetal protein, *BCLC* Barcelona clinic liver cancer, *TNM* tumor node metastasis, *AJCC* American Joint Committee On Cancer, *HCC* hepatocellular carcinoma, *GLDC* glycine decarboxylase

*P < 0.05

### Cell culture and transfection

The human HCC cell line PLC was obtained from the American Type Culture Collection biobank. The Huh7 cell line was obtained from the Japanese Collection of Research Biosources. MHCC97L and HCCLM3 were cultured as described^29,30^. The HCC cell lines were authenticated using an STR Multi-amplification Kit (Microread TM21 ID System) for DNA typing by Microread (Beijing, China) in July 2017. GLDC and miR-30d-5p were transfected as previously described^20^. MiR-30d-5p mimics, inhibitors, and their corresponding controls were purchased from GenePharma (Shanghai, China). The cells were grown in Dulbecco’s modified Eagle’s medium (DMEM; Invitrogen, Carlsbad, CA) with 10% fetal bovine serum (FBS; Invitrogen, Carlsbad, CA) and 1% penicillin/streptomycin (Invitrogen, Carlsbad, CA) at 37 °C under 5% CO_2_.

### Immunohistochemistry (IHC) staining

The paraffin-embedded tissue samples were cut into 5 μm thick sections. The sections were deparaffinized in xylene three times for 5 min each, rehydrated in graded alcohols, incubated in 3% hydrogen peroxide for 30 min, and boiled in 10 mM citrate buffer (pH 6.0) for antigen retrieval. Then, the sections were incubated overnight at 4 °C with a GLDC antibody (Sigma-Aldrich, USA). The primary antibodies were used for visualization of GLDC protein expression levels with the Polink-1 HRP DAB detection system (ZSGB-Bio, Beijing, China).

### mRFP-EGFP-LC3B assay

HCC cells (1 × 10^4^ per well) were seeded in 24-well plates with microscope cover slips and cultured for 24 h before being transient transfected with mRFP-EGFP-LC3B (Addgene, Watertown, MA, USA, plasmid #21074; deposited by Tamotsu Yoshimori) using Lipofectamine 3000 (Invitrogen, Carlsbad, USA) for 36 h, or starved with Hank’s balanced salt solution (HBSS, Invitrogen, Carlsbad, CA) for different lengths of time. Following treatment, cells were fixed with 4% paraformaldehyde in phosphate-buffered saline (Solarbio, Beijing, China). Cells with green puncta (GFP-LC3B^+^) or red puncta (mRFP-LC3B^+^) or yellow puncta (GFP^+^ mRFP^+^) were detected by confocal microscopy.

### Western blot analysis

The same amount of total cell lysate was prepared for western blotting as previously described^31^. Antibodies against p62 (ab109012, Abcam, Cambridge, UK), β-actin (YM3028, ImmunoWay Biotechnology, Plano, TX, USA), LC3B (#3868, Cell Signaling Technology, Beverly, MA, USA), and GLDC (#12794, Cell Signaling Technology, Beverly, MA, USA) were used. The blots were subsequently developed by enhanced chemiluminescence (Millipore, Burlington, MA, USA) using a horseradish peroxidase-conjugated secondary antibody (Santa Cruz Biotechnology, Dallas, TX, USA)).

### RNA extraction and Quantitative real time polymerase chain reaction (qRT-PCR)

Total RNA was extracted using TRIzol reagent (Invitrogen, CA) according to the manufacturer’s instructions. Reverse transcription reactions were performed with 1 μg of total RNA using FastQuant RT kit (TIANGEN Biotech, Beijing, China). The sequence of the miR-30d-5p stem loop is 5ʹ-GTCGTATCCAGTGCAGGGTCCGAGGTATTCGCACTGGATACGACCTTCCA-3ʹ. GLDC expression was quantified using a SYBR qPCR Kit (TIANGEN Biotech) according to the manufacturer’s instructions. All samples were run in triplicate. The endogenous RNA reference gene used was 18s ribosomal RNA. The relative expression levels were evaluated using the 2^−ΔΔCt^ method^20^. Primers are listed in Supplementary Table S1.

### Luciferase reporter assay

A 70-bp fragment of the GLDC 3ʹ-UTR or 3ʹ-UTR mutant sequence was cloned into the pmirGLO dual-luciferase reporter plasmids (Promega, Madison, WI, USA). The Huh7 and PLC cells were cultured on 24-well tissue culture plates at a density of 3 × 10^4^ cells per well, followed by co-transfection with the reporter constructs together with miR-30d-5p-mimic, miR-30d-5p inhibitor, or their corresponding controls using Lipofectamine 3000.

### Migration and invasion assays

Migration and invasion assays were performed using 24-well Transwell chambers containing polycarbonate membranes with 8-μm pores (Corning, Tewksbury MA, USA). For the invasion assays, the membrane was coated with Matrigel (BD Biosciences, San Jose, CA, USA). Serum-starved cells (2 × 10^5^) were added to the upper chamber and incubated in serum-free medium. Then, 600 μl of DMEM with 10% FBS was added to the lower chamber. Cells were incubated at 37 °C under 5% CO_2_ for 20 h. After that, non-migrating or non-invasive cells on the upper membrane surface were removed with a cotton swab, whereas the migrating and invasive cells on the under surface were fixed and stained. The number of migrating and invasive cells were counted microscopically.

### In vivo metastasis assays

Male BALB/c-nude mice (5-week-old, Chinese Academy of Sciences, Beijing, China) were used for the intrahepatic metastasis assays^20^. Briefly, 2 × 10^6^ cells were suspended in 20 μl of serum-free DMEM and 20 μl of Matrigel for each mouse (*n* = 6 mice for each cell line). Through an 8-mm midline incision in the upper abdomen under anesthesia, cells were orthotopically inoculated in the left hepatic lobe by a microsyringe. After 6 weeks, mice were sacrificed, and their livers were dissected, and fixed with 4% paraformaldehyde for following standard histological examination. The experimental protocols were evaluated and approved by the Tianjin Medical University Animal Care and Use Committee.

### Statistical analysis

Clinicopathological correlations were analyzed by Pearson’s chi-square test. Overall survival (OS) and disease-free survival (DFS) were calculated by Kaplan–Meier survival analysis and log-rank tests. The expression correlation between GLDC and miR-30d-5p was determined using Pearson’s correlation coefficient. The Student’s *t*-test was used for comparison between two groups, and one-way analysis of variance was used for analysis among groups. Data are presented as the mean ± standard deviation. SPSS17.0 software (SPSS, Chicago, IL, USA) was used for all data analyses, and *P*-values <0.05 were considered statistically significant.

## Results

### GLDC is an independent prognostic factor for HCC patients

**Fig. 1:**
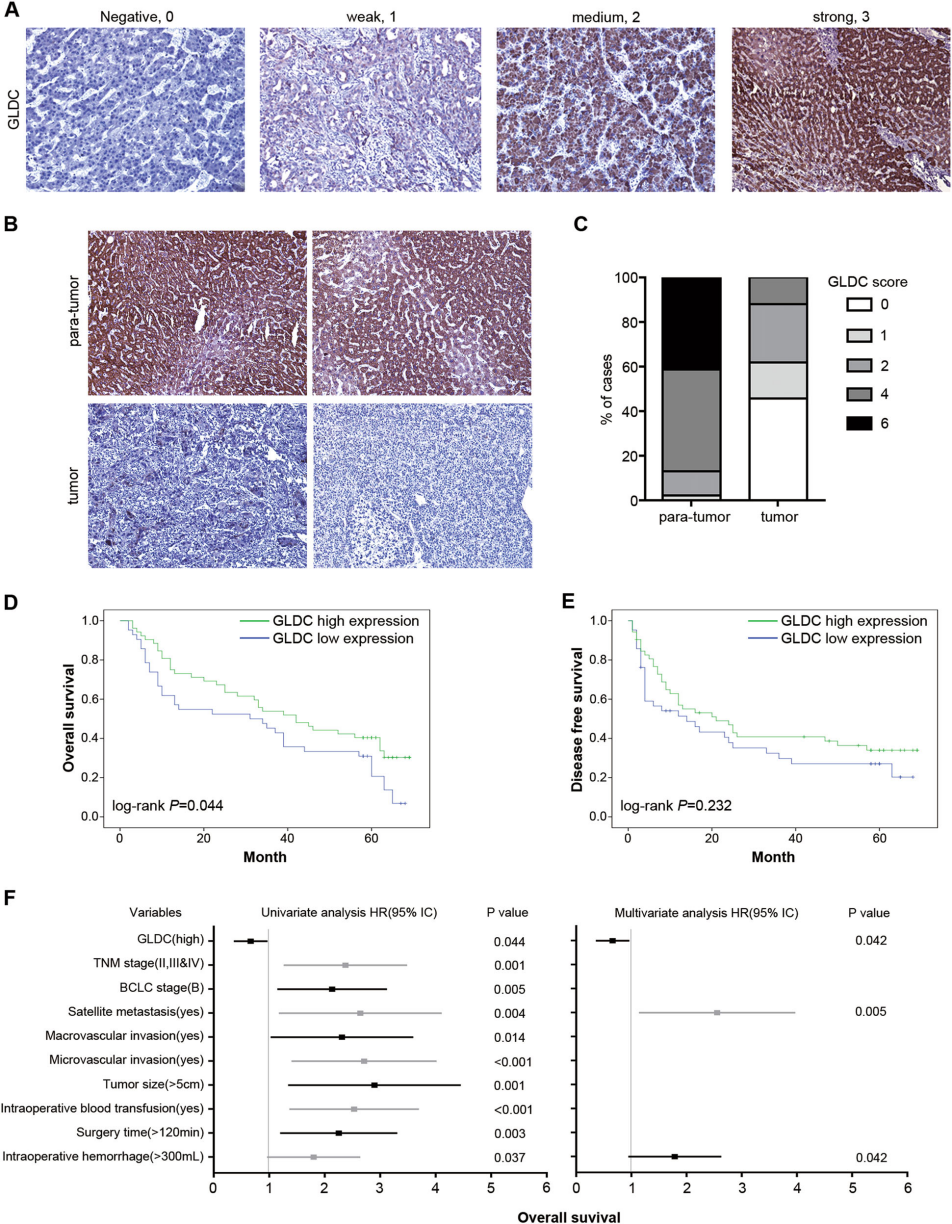
Glycine decarboxylase (GLDC) is an independent prognostic factor for hepatocellular carcinoma (HCC) patients. **a** Representative staining patterns of GLDC in different staining intensity (magnifications: ×100). **b** Representative immunohistochemistry (IHC) analysis of GLDC expression in 94 paired HCC tissue samples. (magnifications: ×100). **c** Percentage chart showed the ratio of cases with GLDC staining overall scores in HCC tissues and para-tumor tissues. **d** Kaplan–Meier’s analyses of correlation between GLDC expression levels and overall survival (OS). **e** Kaplan–Meier’s analyses of correlation between GLDC expression levels and disease-free survival (DFS). **f** Univariate and multivariate Cox regression analysis of prognostic factors for OS in 94 HCC patients. Hazard ratios (HRs) are presented as the means (95% confidence interval (CI)). Survival curves were compared by a log-rank test, and variables used in the multivariate analysis were chosen by the univariate analysis

Our previous study showed that HCC tumors expressed lower GLDC levels, which was correlated with a poor survival rate of HCC patients in The Cancer Genome Atlas (TCGA) database^20^. To further confirm our findings, we performed IHC analysis of a tissue microarray from a large cohort of HCC clinical specimens (*n* = 94). The staining intensity (I) was categorized by relative intensities of 0 (negative staining), 1 (weak staining), 2 (medium staining), or 3 (strong staining) (Fig. 1a). Stronger GLDC immunostaining was observed in para-tumor tissues compared with tumor tissues (Fig. 1b). The percent of immunopositive cells (P) in each microscope field was categorized as 0 (<10%), 1 (>10% and <50%), and 2 (>50%). An overall score was determined as I × P. Then, GLDC expression level in tumor tissue was scored as 0, 1, 2, 4, and 6 in 42, 15, 24, 11, and 2 samples, respectively. By contrast, in para-tumor tissue, GLDC expression level was score as 0, 1, 2, 4, and 6 in 0, 2, 10, 42, and 40 samples, respectively (Fig. 1c). These results further showed lower GLDC expression levels in tumor tissues than corresponding para-tumor tissues.

We also examined the relationship between downregulation of GLDC with outcomes and clinicopathological characteristics of HCC patients. GLDC expression level in tumor tissues was categorized by overall score as low (overall score 0) and high (overall score >0) expression groups. Kaplan–Meier analysis showed that lower expression levels of GLDC in tumors were significantly associated with worse OS (Fig. 1d), whereas recurrence-free survival (DFS) did not show significant differences (Fig. 1e). Lower expression of GLDC was detected in 42 of 94 HCC samples (Table 1). Downregulation of GLDC was associated with tumor shape, lymph node metastasis, BCLC stage, and TNM stage. By univariate Cox regression analyses, GLDC downregulation, TNM stage, BCLC stage, satellite metastasis, macrovascular invasion, microvascular invasion, tumor size, intraoperative blood transfusion, surgery time, and intraoperative hemorrhage were significantly correlated with worse OS (Fig. 1f, left panel). In multivariate analyses, GLDC downregulation, satellite metastasis, and intraoperative hemorrhage were independent prognostic factors for poor OS in HCC patients (Fig. 1f, right panel). Altogether, these results further show that the downregulation of GLDC is an independent prognostic factor for HCC patients and might play an important role in HCC metastasis.

### GLDC downregulation reduces cell autophagy

Redox-dependent autophagy largely relies on the magnitude and the rate of ROS generation^15^. Our previous study reported that GLDC downregulation induced a decrease in the GSH/GSSG ratio, suggesting an increase in ROS levels. Moreover, NAC, an antioxidant, reversed GLDC-knockdown induced cell migration and invasiveness in HCC cells^20^. Therefore, we examined the ability of GLDC to modulate autophagy in HCC cells. First, GLDC was stably knocked down in PLC and Huh7 cells or overexpressed in MHCC97L and HCCLM3 cells. The efficiency of knockdown or overexpression was confirmed by qRT-PCR analysis and western blot analysis (Supplementary Figure S1).

Microtubule-associated protein 1 light chain 3 (LC3) is a suitable marker for autophagy^32^. A marked decrease in LC3 puncta formation was observed in GLDC-knockdown PLC and Huh7 cells compared with the corresponding control cells (Supplementary Figure S2A). By contrast, there was an increase of LC3 puncta formation in GLDC-overexpressing MHCC97L and HCCLM3 cells compared with the corresponding control cells (Supplementary Figure S2B). The ratio of LC3-II to LC3-I (LC3-II/LC3-I) is an important indicator of autophagy activity, with a higher LC3-II/LC3-I ratio reflecting higher autophagy activity. The cytoplasmic form LC3-I (18 kDa) is generated from LC3 cleavage and is converted to phagophore-associated LC3-II (16 kDa)^33^. SQSTM1/p62 (sequestosome 1) links ubiquitinated substrates and LC3-II, and is degraded in autolysosomes. With activation of autophagic flux, p62 degradation is increased and p62 expression levels is decreased^34,35^. We found that GLDC downregulation increased p62 expression and reduced LC3-II/LC3-I ratio in PLC and Huh7 cells (Supplementary Figure S2C). By contrast, GLDC overexpression decreased p62 expression and increased the LC3-II/LC3-I ratio in MHCC97L and HCCLM3 cells (Supplementary Figure S2D). These results indicate that GLDC might be involved in autophagy regulation.

**Fig. 2:**
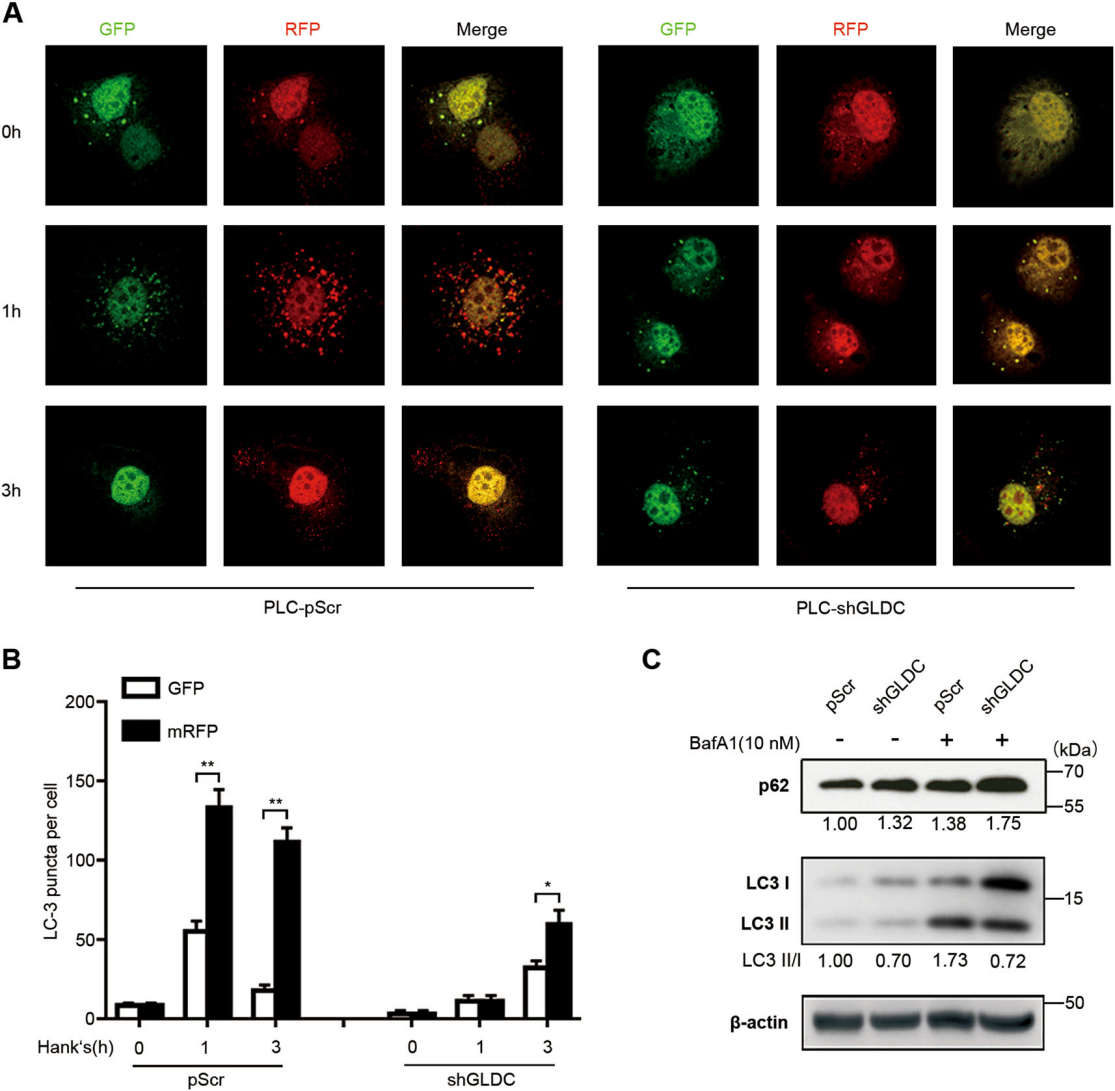
Glycine decarboxylase (GLDC) downregulation reduces autophagy. **a** Representative immunofluorescence images of GLDC knockdown PLC and its corresponding control cells with incubation in Hank’s balanced salt solution (HBSS) for 0, 1, and 3 h, after 48 h of mRFP-GFP-LC3 plasmid transfection. **b** Histogram of LC3 puncta counts in GLDC knockdown PLC and its corresponding control cells. **c** Western bolts of LC3 and p62 in GLDC knockdown PLC cells and its corresponding control cells. All experiments were repeated three times and the representative results were shown. *P < 0.05, **P < 0.005

**Fig. 3:**
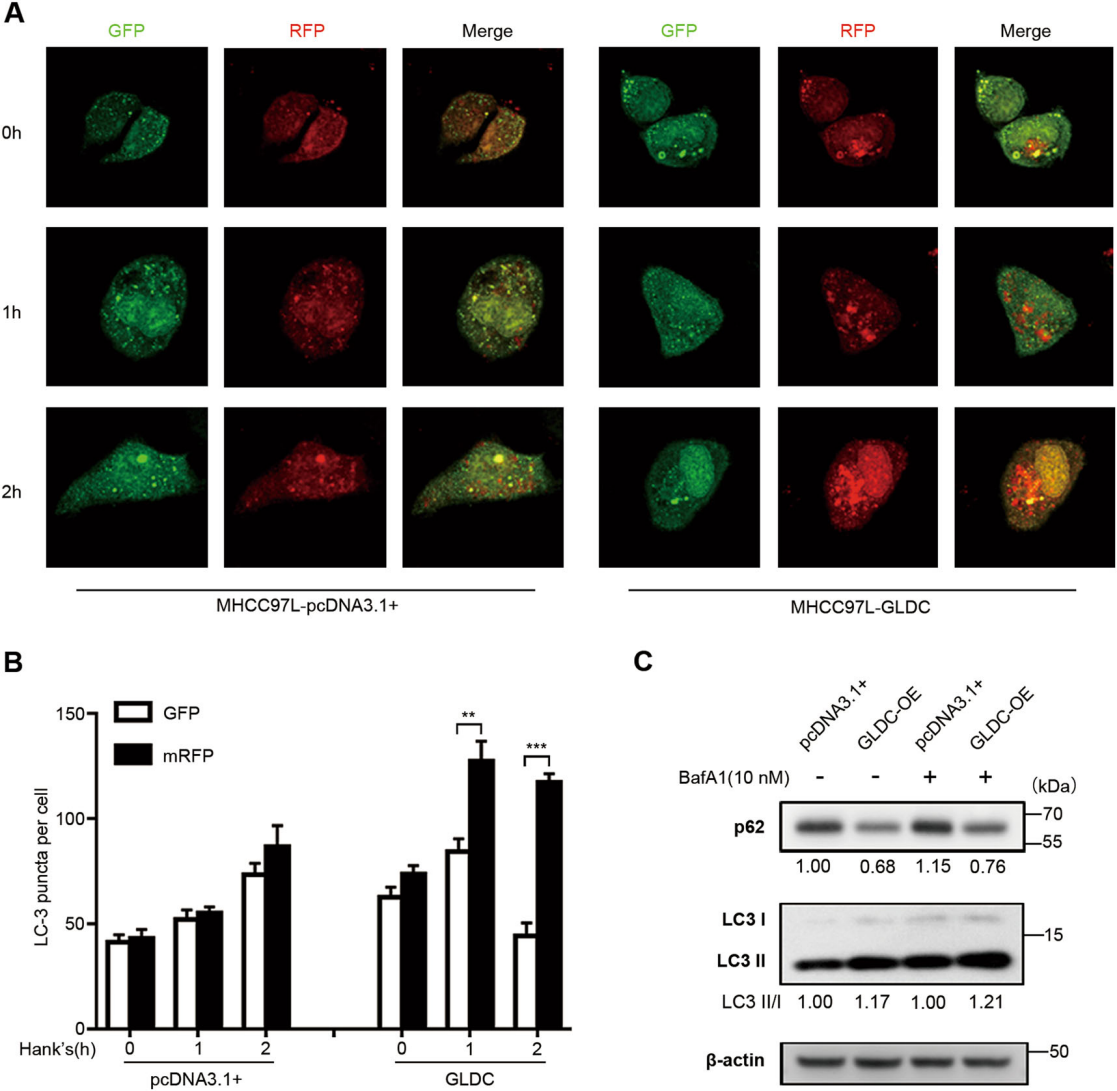
Glycine decarboxylase (GLDC) upregulation induces autophagy. **a** Representative immunofluorescence images of GLDC-overexpressing MHCC97L and its corresponding control cells with incubation in Hank’s balanced salt solution (HBSS) for 0, 1, and 2 h, after 48 h of mRFP-GFP-LC3 plasmid transfection. **b** Histogram of LC3 puncta counts in GLDC-overexpressing MHCC97L and its corresponding control cells. **c** Western bolts of LC3 and p62 in GLDC-overexpressing MHCC97L cells and their corresponding control cells. All experiments were repeated three times and the representative results were shown. **P < 0.005, ***P < 0.0005

Next, we detected the role of GLDC in stress-induced autophagy. Upon nutrient starvation, GLDC downregulation inhibited cell autophagy (Fig. 2a, b). An autophagy inhibitor, bafilomycin A1 (BafA1), was used to treat GLDC-knockdown PLC cells. GLDC downregulation increased p62 expression and reduced LC3-II/LC3-I ratio, effects that was further augmented by BafA1 in PLC cells (Fig. 2c). Whereas, GLDC overexpression increased cell autophagy (Fig. 3a, b). BafA1 treatments inhibited effects of the decreased p62 expression and the increased LC3-II/LC3-I ratio in GLDC-overexpressing MHCC97L cells (Fig. 3c). Taken together, these results indicate that autophagic flux was inhibited with GLDC downregulation in HCC cells.

### GLDC is a downstream target of miRNA-30d-5p

**Fig. 4:**
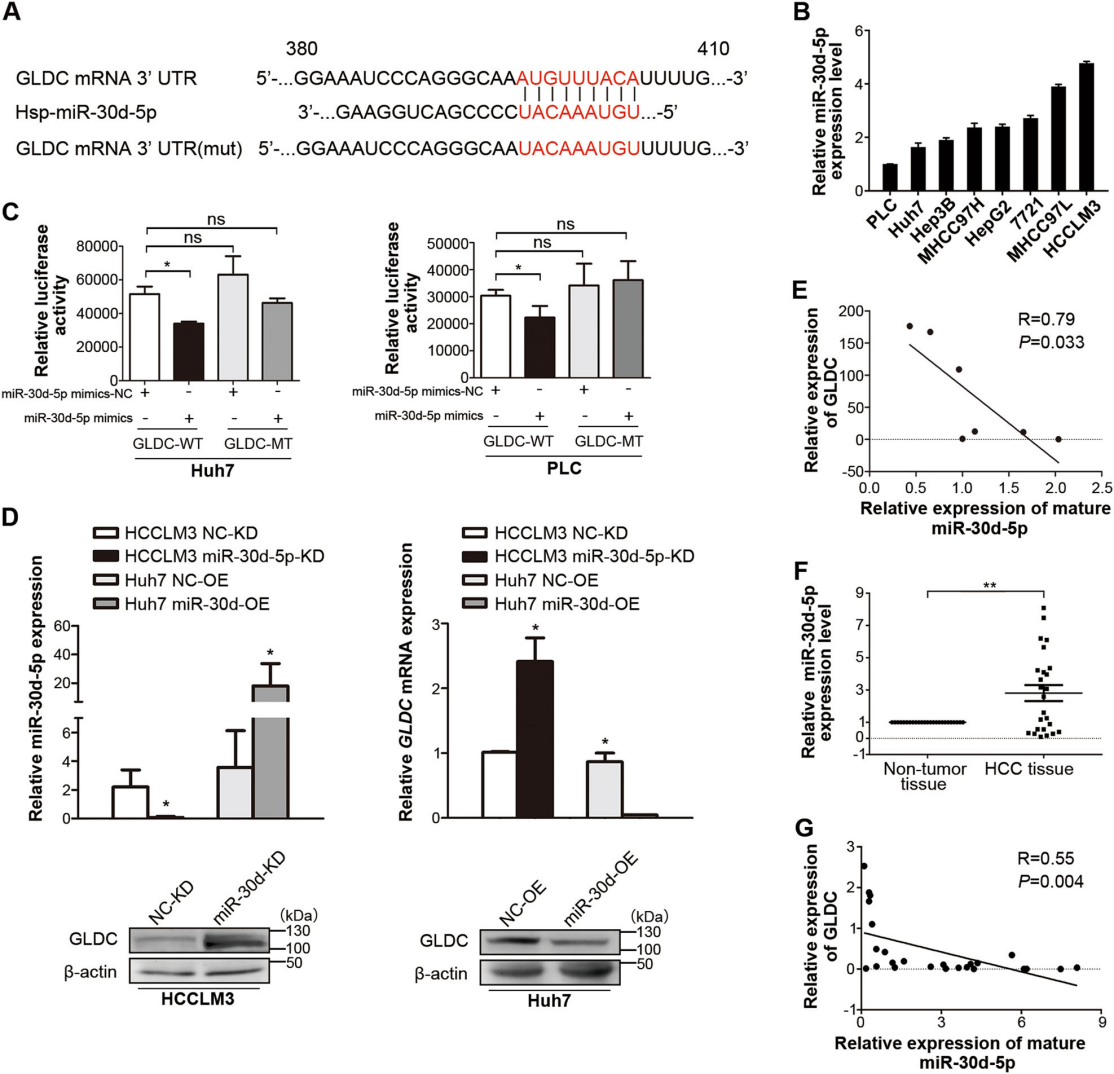
Glycine decarboxylase (GLDC) is a downstream target of miRNA-30d-5p. **a** Predicted binding site between 3ʹ-UTR of GLDC mRNA and miR-30d-5p. **b** qPCR arrays of miR-30d-5p using a number of hepatocellular carcinoma (HCC) cell lines. **c** Luciferase reporter gene assays using Huh7 and PLC cells co-transfected with miR-30d-5p mimics and GLDC wild-type 3ʹ-UTR or its mutant 3ʹ-UTR. **d** Expression levels of miR-30d-5p and GLDC in HCCLM3 cells transfected with miR-30d-5p inhibitor or in Huh7 cells transfected with miR-30d-5p mimics by qPCR (upper panel) and WB (below panel). Data represent the means ± SD (*P < 0.05, by Student’s *t*-test). **e** The correlation between GLDC and miR-30d-5p expressions in HCC cells (Pearson’s correlation coefficient, *R* = 0.79, *P = 0.033). **f** qPCR analysis of miR-30d-5p expression in 25 paired HCC clinical samples. Data represent the means ± SD (**P < 0.005, by Student’s *t*-test). **g** The correlation between GLDC and miR-30d-5p expressions in 25 paired HCC clinical samples (Pearson’s correlation coefficient, *R* = 0.55, *P* = 0.004)

Our previous study showed that GLDC expression was significantly downregulated in the malignant HCC cell lines, MHCC97L, and HCCLM3 cells, compared with Huh7 cells^20^. A microRNA array has also been analyzed for Huh7, MHCC97L, and HCCLM3 cells (data not shown). Among those differentially expressed microRNAs in MHCC97L and HCCLM3 cells compared with Huh7 cells, miR-30d-5p was the only upregulated microRNA that was predicted to be a potential regulator of GLDC based on bioinformatic analysis, including TargetScan, microRNA, PicTar, and RNAhybrid (Fig. 4a). qRT-PCR confirmed a remarkable increase in miR-30d-5p in MHCC97L and HCCLM3 cells compared with Huh7 cells (Fig. 4b). MiR-30d-5p is known to promote cell autophagy, migration, and invasion in cancer cells^36–38^. Thus, we chose to investigate the relationship between miR-30d-5p and GLDC for subsequent study.

Luciferase reporter gene plasmids containing GLDC wild-type 3ʹ-UTR or its mutant 3ʹ-UTR were constructed (Fig. 4a). The results showed that miR-30d-5p overexpression suppressed GLDC 3ʹ-UTR luciferase activities in PLC and Huh7 cells (*P* < 0.05). By contrast, mutation of the miR-30d-5p binding site in the 3ʹ-UTR GLDC luciferase vector abolished the suppressive effects of miR-30d-5p (Fig. 4c). Moreover, inhibition of miR-30d-5p resulted in upregulation of GLDC expression at the mRNA and protein levels in HCCLM3 cells. By contrast, overexpression of miR-30d-5p significantly suppressed GLDC expression in Huh7 cells (Fig. 4d).

Our previous study examined the expression levels of GLDC in a number of HCC cells and 25 paired HCC samples^20^. To study the human relevance of GLDC and miR-30d-5p, we examined the expression levels of miR-30d-5p in those HCC cells (Fig. 4b). The expression of miR-30d-5p was inversely correlated with GLDC in HCC cells (Fig. 4e, Pearson correlation coefficient *R* = 0.79, *P* < 0.05). In addition, miR-30d-5p expression was upregulated in HCC tissues compared with the corresponding para-tumor tissues (Fig. 4f, *P* < 0.005). Correlation analysis of the HCC patient data further supported that GLDC expression was inversely correlated with miR-30d-5p expression (Fig. 4g, Pearson correlation coefficient *R* = 0.55, *P* < 0.005). Taken together, those results indicate that GLDC is likely to be a downstream target gene of miR-30d-5p.

### GLDC regulates autophagy and invasiveness through epigenetic silencing by miR-30d-5p

**Fig. 5:**
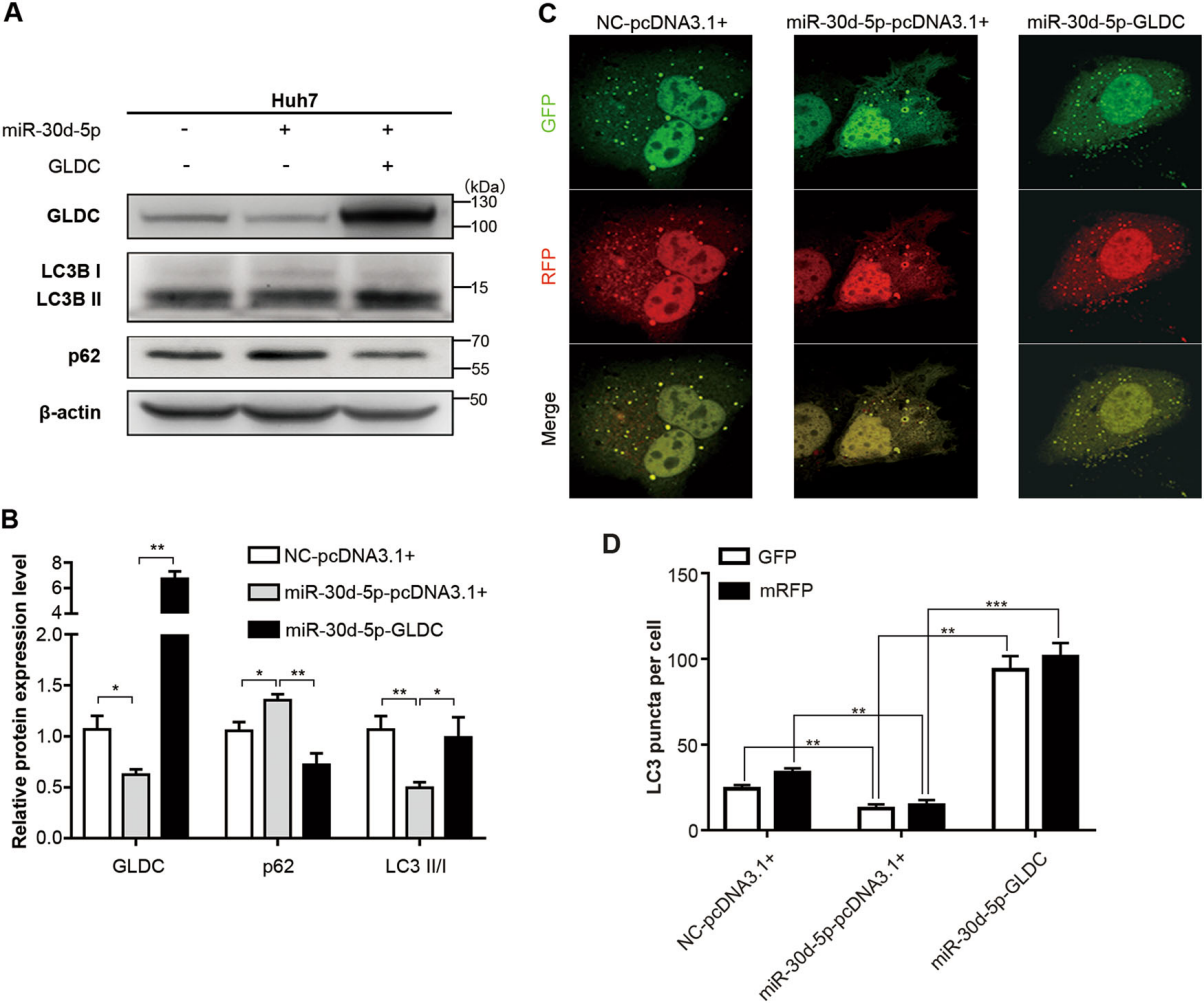
Glycine decarboxylase (GLDC) regulates autophagy through epigenetic silencing by miR-30d-5p. **a** Western bolts of GLDC, LC3, and p62 in Huh7 cells co-transfected with miR-30d-5p mimics and GLDC expression construct. **b** Histogram showed the relative intensity of GLDC versus β-actin, p62 versus β-actin, and LC3-II/I in Huh7 cells co-transfected with miR-30d-5p mimics and GLDC expression construct (*P < 0.05, **P < 0.005). **c** Representative immunofluorescence images of Huh7 cells with incubation in Hank’s balanced salt solution (HBSS) for 1 h, after 48 h of co-transfection of miR-30d-5p mimics and GLDC expression construct and 24 h of mRFP-GFP-LC3 plasmid transfection. **d** Histogram of LC3 puncta counts in Huh7 cells co-transfected with miR-30d-5p mimics and GLDC expression construct (*P < 0.05, **P < 0.005, ***P < 0.0005)

Next, we determined whether GLDC plays a role in HCC cell autophagy via miR-30d-5p regulation. Rescue experiments were performed by co-transfection with miR-30d-5p mimics and a GLDC expression construct (Fig. 5a and Supplementary Figure S3). Overexpression of GLDC increased the ratio of LC3-II/LC3-I and decreased p62 expression level in miR-30d-5p-overexpressing Huh7 cells (Fig. 5a, b). Immunofluorescence assays showed that GLDC overexpression increased LC3 puncta formation that were otherwise inhibited by transfection with miR-30d-5p alone (Fig. 5c, d).

**Fig. 6:**
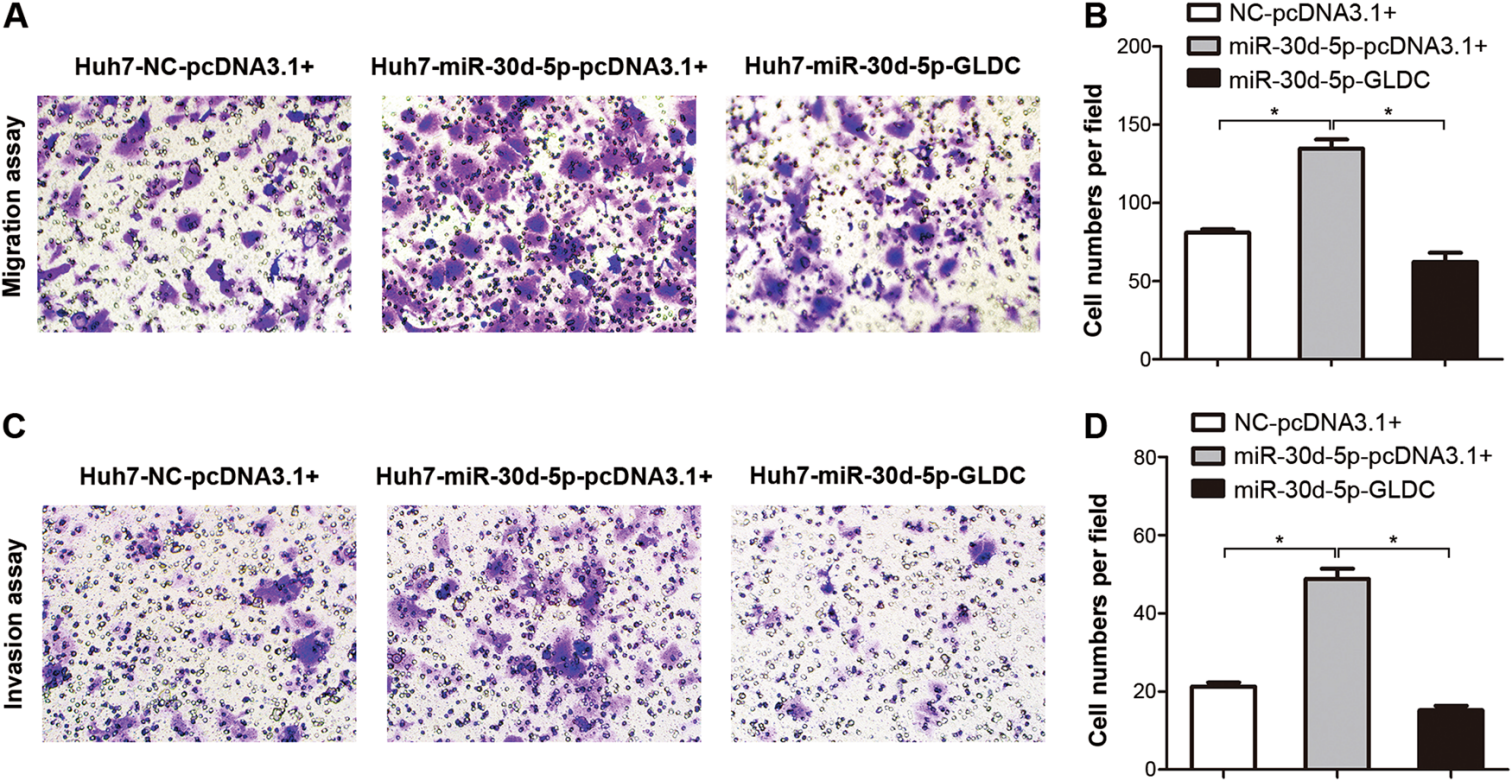
Glycine decarboxylase (GLDC) regulates migration and invasiveness through epigenetic silencing by miR-30d-5p. **a** Transwell chamber assays using Huh7 cells co-transfected with miR-30d-5p mimics and GLDC expression construct. **b** Representative images of the migratory cells (left panel), magnification: ×200. Histogram of the numbers of migratory (right panel, *P < 0.05). **c** Matrigel invasion assays using Huh7 cells co-transfected with miR-30d-5p mimics and GLDC expression construct. Representative images of the invading cells (left panel), magnification: ×200. **d** Histogram of the numbers of invasion (right panel, *P < 0.05)

We further examined the role of GLDC in miR-30d-5p-dependent cell migration and invasion. Overexpression of miR-30d-5p significantly enhanced cell migration and invasion in Huh7 cells (Supplementary Figure S4A). By contrast, downregulation of miR-30d-5p markedly decreased cell migration and invasion in HCCLM3 cells (Supplementary Figure S4B). The restoration of GLDC significantly impaired cell migration and invasiveness initiated by miR-30d-5p (Fig. 6). Taken together, the results suggest that GLDC is able to regulate cell autophagy and invasiveness through epigenetic silencing by miR-30d-5p.

### GLDC overexpression inhibits intrahepatic metastasis in vivo

**Fig. 7:**
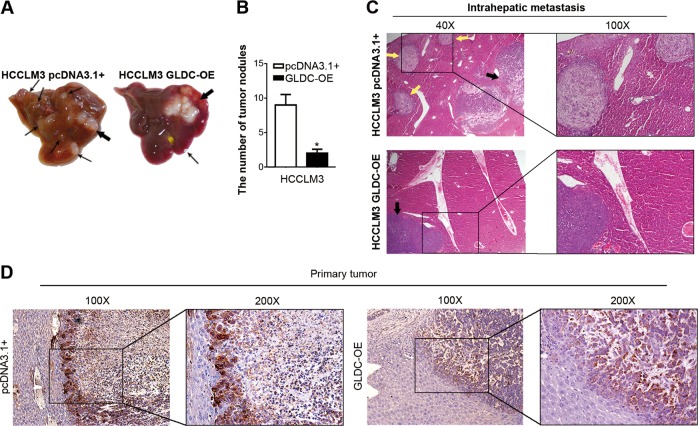
Glycine decarboxylase (GLDC) overexpression inhibits intrahepatic metastasis in vivo. **a** Representative images showing the orthotopic transplanted tumor (marked with bold blank arrows) and the intrahepatic metastases (marked with thin blank arrows) in the orthotopic mouse models transplanted with GLDC-overexpressing HCCLM3 cells and the corresponding control cells. **b** Histogram showing the surface tumor nodules (*n* = 5, *P < 0.05, by Student’s *t*-test). **c** Representative hematoxylin and eosin (H&E) staining images of the orthotopic transplanted tumor (marked with bold blank arrows) and the intrahepatic metastasis (marked with bold yellow arrows). **d** Representative immunohistochemistry (IHC) images of expressions of p62 in the orthotopic transplanted tumors transplanted with GLDC-overexpressing HCCLM3 cells and the corresponding control cells

Our previous study determined the metastatic relevance of GLDC in vivo^20^. Therefore, we further examined the autophagy relevance of GLDC in vivo. An orthotopic HCC mouse model was established by intrahepatic inoculation. Mice injected with GLDC-overexpressing HCCLM3 cells had fewer intrahepatic metastases compared with the corresponding control group (9.00 ± 1.53 versus 2.00 ± 0.58, *P* < 0.05, Fig. 7a, b). Histologic analyses confirmed the finding of fewer intrahepatic metastases in mouse liver transplanted with GLDC-overexpressing HCCLM3 cells compared with the corresponding control cells (Fig. 7c). Moreover, p62 expression was markedly decreased in the primary tumor of mice injected with GLDC-overexpressing cells compared with those transplanted with the corresponding control cells (Fig. 7d). These results further confirm that GLDC inhibits HCC metastasis via cell autophagy.

## Discussion

The pathological relevance and significance of autophagy inhibition in cancer cell metastasis is gaining experimental support^11^. Autophagy is correlated with ROS and thiol redox state in carcinoma cells^15,39,40^. Our previous GLDC study showed that downregulation of GLDC raised ROS levels and reduced GSH/GSSG ratio in HCC cells. Furthermore, we made the novel discovery that cofilin stabilization in the process of ROS-enhanced cell migration is a mechanism by which downregulation of GLDC promotes cell migration^20^. Here, we further report that GLDC is an independent prognostic factor for HCC patients as assessed by Kaplan–Meier analysis and Cox regression analysis. Our data suggest that GLDC upregulation diminished miR-30d-5p-reduced autophagy and consequently inhibited HCC progression.

Tumor cells acquire metabolism rewiring, which confers many advantages, including maintaining redox balance^41^. Under oxidative stress, tumor cells have more aggressive phenotypes^39^. Moreover, many anticancer drugs increase ROS production, which is conductive to induction of drug resistance in tumor cells^42^. Activation of autophagy could reduce ROS levels and limit the tumor-promoting effects of ROS^18,43^. Increased ROS levels and enhanced HCC progression have been observed in GLDC knockdown HCC cells^20^. Therefore, we surmise that the enhanced HCC progression caused by the increased ROS levels in GLDC knockdown HCC cells is attributable to the inhibition of autophagy. Indeed, our present observation of increased p62 expression in HCC cells in which GLDC has been knocked down is consistent with previous study, wherein high levels of p62 in HCC are proposed to be associated with suppression of autophagy, resulting in activation of the stress-responsive transcription factor Nrf2^43^. Our mouse model analysis also showed that lower expression levels of p62 were observed in the primary tumors of mice injected with GLDC-overexpressing HCC cells. Moreover, the LC3-II/LC3-I ratio was increased with upregulation of GLDC in HCC cells. The results, therefore, suggest that metastatic HCC cells have lower expression levels of GLDC to protect themselves from cellular autophagy and sustain their metastatic capacity.

Of note, we suggest a novel mechanism herein by which GLDC inhibited HCC metastasis through induction of autophagy. GLDC belongs to the GCS that consumes glycine^22^. GCS function is indicated by its subcellular localization in the malaria parasite *Plasmodium falciparum*. H-protein, one of the GCS protein subunits, is localized to the mitochondrion in *P. falciparum*^44^. The human GCS is localized in the inner mitochondrial membranes of brain, kidney, and liver^45^. Our results also showed that GLDC located in the mitochondria of HCC cells (data not shown). There has been evidence to link mitochondrial activity and autophagy^46^. Mitochondrial redox state is one of the dysregulators of mitochondrial function^47^. The fact that GLDC decreases ROS production and induces GSH/GSSG ratio in HCC cells^20^ and the present report, suggests that the decreased ROS production in GLDC-overexpressing cells is linked with the increased autophagic signal transduction in HCC cells, which is worthy of further elucidation.

Upregulation of miR-30d promotes invasion and migration of HCC cells in vitro and in vivo^36^. Our results also showed that miR-30d-5p enhanced HCC progression, which is consistent with a previous report^36^. Many microRNAs are reported to be involved in autophagic regulation, including miR-30d^37,48^. MiR-30d impairs cellular autophagy and suppresses expressions of multiple core autophagy genes, such as ATG2B, ATG5, ATG12, BECN1, and BNIP3L^37^. MiR-30d-5p also suppresses antioncogenic gene expression in HCC. For example, Galphai2, a metastasis suppressor, has been identified as a direct and functional target of miR-30d in HCC^36^. Our results showed that GLDC was also a post-transcriptional target of miR-30d-5p in HCC cells. GLDC expression could be silenced by miR-30d-5p. GLDC overexpression inhibited migration and invasion via an increase in cellular autophagy. This effect was reduced by miR-30d-5p transfection. Therefore, our results emphasized the importance of miR-30d-5p as a potential therapeutic target for HCC treatment.

Cancer cells often utilize aerobic glycolysis instead of mitochondrial oxidative phosphorylation to generate ATP and biosynthetic intermediates for rapid growth^49^. Published work has highlighted that cell migration is supported also by ATP produced by glycolysis, rather than by mitochondrial respiration^50,51^. Enhanced ATP yield can be produced by glycolysis and increase cell migration in the presence of mitochondrial dysfunction^50^. In the light of our previous data showing the increased ROS production in GLDC knockdown HCC cells, mitochondrial activity might be associated with impaired autophagy in GLDC knockdown HCC cells. In the other aspect, GLDC is coupled to the serine biosynthesis^23^. Serine hydroxymethyltransferase (SHMT) catalyzes the reversible conversion of serine to glycine. The mitochondrial SHMT2, but not cytosolic SHMT1, is highly expressed in cancer cells and tissues and promotes cancer tumorigenesis^25,52^. Cells with high expression of SHMT2 would be sensitive to downregulation of GLDC because excess glycine is converted into toxic metabolites that impair cell growth^22^. Studies suggest that autophagy promotes cancer cell growth^53^. Liver-specific deletion of *Atg7* reduced tumor growth^54^. Here we showed that the autophagic flux is decreased with downregulation of GLDC. Therefore, the growth arrest in cells with high SHMT2 levels and GLDC suppression could be partly due to autophagy failure. Further investigation is warranted to clarify the potential mechanistic roles of GLDC in cellular autophagy.

The function of autophagy is suggested to be context dependent in tumor development^55–59^. In light of the studies show that GLDC expression is also tumor-type specific, the effect of GLDC on cellular autophagy might be tumor-type specific. Furthermore, miR-30d is found to be downregulated and functions as a tumor suppressor in some other types of cancers, such as NSCLC^26^ and esophageal squamous cell carcinoma^59^. Notably, GLDC acts as oncogene in the tumorigenesis of NSCLC cells^26^. Till now, GLDC inhibitor is not available. A recent study used steric hindrance antisense oligonucleotide to downregulate GLDC expression and observed its antitumor effect in lung cancer cell lines^27^. Thus, the function of GLDC, and relationship between GLDC and miR-30d-5p should be carefully considered in different tumors.

In summary, we have identified that GLDC is an independent factor for predicting prognosis in HCC patients. GLDC is able to regulate cell autophagy and invasiveness through epigenetic silencing by miR-30d-5p in HCC cells. Our findings provide further understanding of GLDC function relevant to HCC progression. Investigation of GLDC may provide novel biomarker candidates for HCC progression.

## Supplementary information

Figure S1

Figure S2

Figure S3

Figure S4

Supplementary file
