# Supplementary figures and images for: Glycine decarboxylase induces autophagy and is downregulated by miRNA-30d-5p in hepatocellular carcinoma
Source: Cell Death Dis. 2019 Feb 25;10(3):192. doi: 10.1038/s41419-019-1446-z (PMC6389915; doi:10.1038/s41419-019-1446-z)

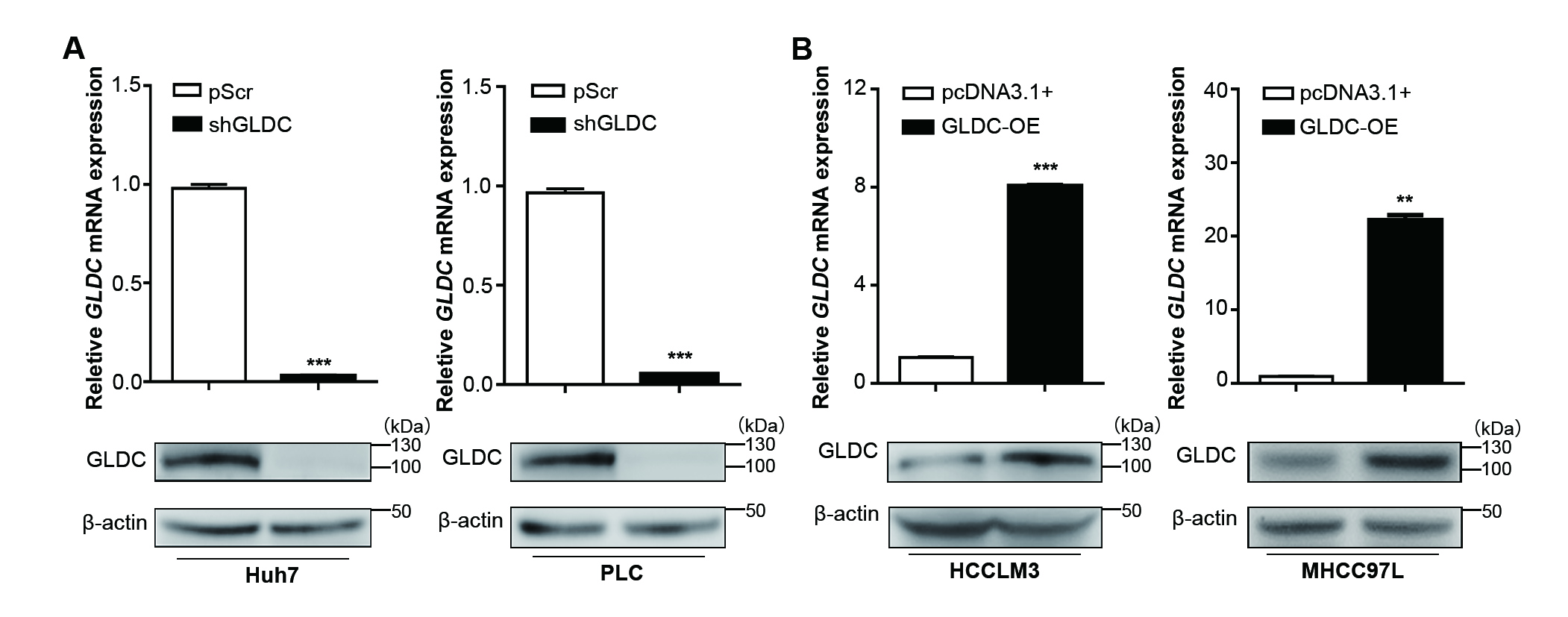

Supplement: Supplementary file 1 — Figure S1 [file 41419_2019_1446_MOESM1_ESM.tif]

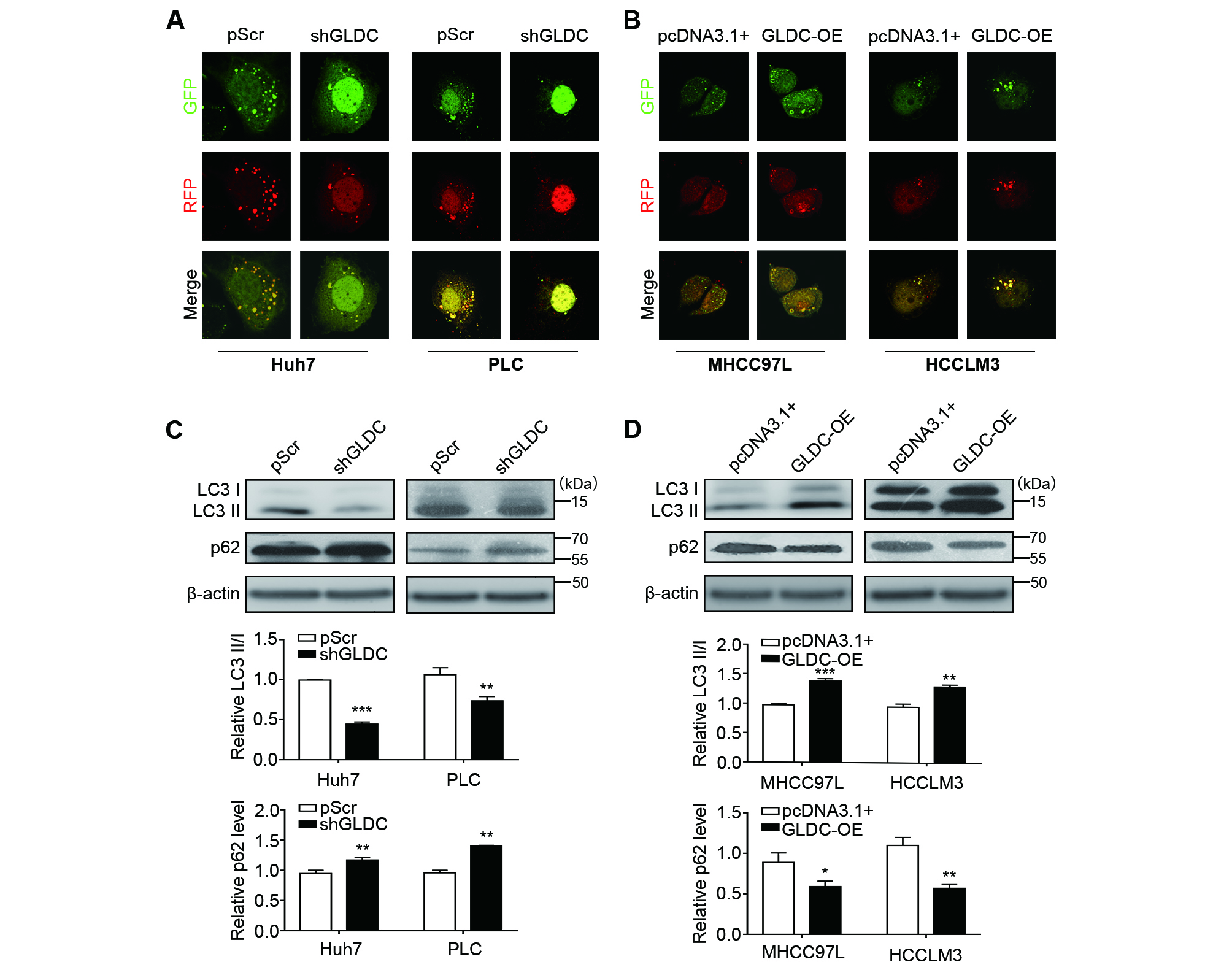

Supplement: Supplementary file 2 — Figure S2 [file 41419_2019_1446_MOESM2_ESM.tif]

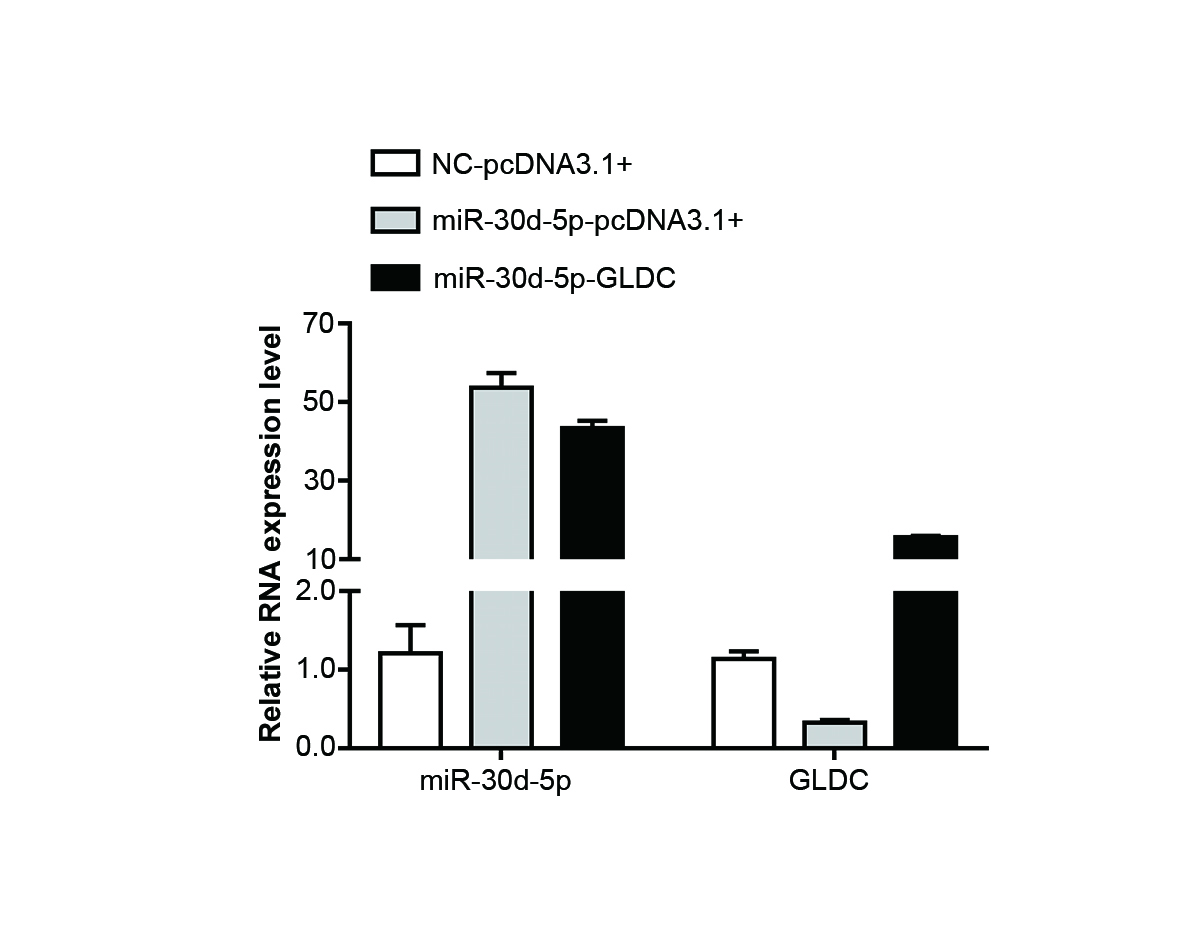

Supplement: Supplementary file 3 — Figure S3 [file 41419_2019_1446_MOESM3_ESM.tif]

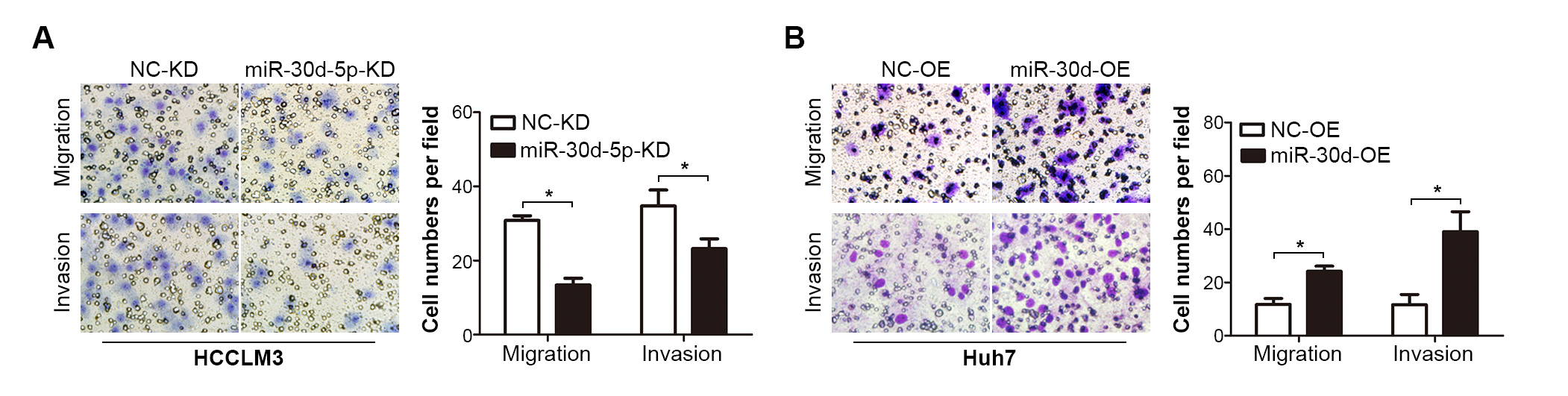

Supplement: Supplementary file 4 — Figure S4 [file 41419_2019_1446_MOESM4_ESM.tif]
