# Supplementary material for: Glycine decarboxylase induces autophagy and is downregulated by miRNA-30d-5p in hepatocellular carcinoma
Source: Cell Death Dis. 2019 Feb 25;10(3):192. doi: 10.1038/s41419-019-1446-z (PMC6389915; doi:10.1038/s41419-019-1446-z)
Supplement: Supplementary file 5 — Supplementary file [file 41419_2019_1446_MOESM5_ESM.docx]

**Supplementary Table S1**. Sequences of the DNA primers for qRT-PCR.

| Name | Sequence (5’-3’) |
| --- | --- |
| GLDC | Forward: GGTCCTGTAAACATCCCC  Reverse: AGCAATTTCCTGCCGAATGC |
| miR-30d-5p | Forward: TGCGCCCCTTAAGAGCAAAA  Reverse: GTGCAGGGTCCGAGGT |
| 18s-rRNA | Forward: GCTTAATTTGACTCAACACGGGA  Reverse: AGCTATCAATCTGTCAATCCTGTC |
| U6 | Forward: CTCGCTTCGGCAGCACA  Reverse: AACGCTTCACGAATTTGCGT |

**Supplementary Figure Legends**

**Figure S1.** **The modifying efficiency of GLDC expression**

A. qPCR and Western blot analysis using GLDC-knockdown cells and its control cells. B. qPCR and Western blot analysis using GLDC-overexpressing cells and its corresponding control cells.

**Figure S2 GLDC downregulation reduces autophagy**

A. Immunofluorescence analysis of GLDC-knockdown PLC or Huh7 cells and their corresponding control cells after incubation in HBSS for 1h. B. Immunofluorescence analysis of GLDC-overexpressing MHCC97L or HCCLM3 cells and their corresponding control cells after incubation in HBSS for 1h. C. Western bolts of LC3 and p62 in GLDC-knockdown PLC or Huh7 cells and their corresponding control cells (upper panel). Histogram showed the relative intensity of LC3 II verses LC3 I and p62 verses β-actin in GLDC-knockdown PLC or Huh7 cells or their corresponding control cells (below panel, ***P* < 0.005, ****P* < 0.001). D. Western bolts of LC3 and p62 in GLDC-overexpressing MHCC97L or HCCLM3 cells and their corresponding control cells (upper panel). Histogram showed the relative intensity of LC3 II verses LC3 I and p62 verses β-actin in GLDC-overexpressing MHCC97L or HCCLM3 cells and their corresponding control cells (below panel, **P* < 0.05, ***P* < 0.005, ****P* < 0.001).

**Figure S3. The modifying efficiencies of miR-30d-5p and GLDC expressions**

qPCR using Huh7 cells co-transfected with miR-30d-5p mimics and GLDC expression construct.

**Figure S4 miR-30d-5p promotes cell migration and invasion in HCC cells**

A. Transwell-chamber assays and Matrigel invasion assays using HCCLM3 cells transient transfected with miR-30d-5p inhibitor or its negative control. B. Transwell-chamber assays and Matrigel invasion assays using Huh7 cells transient transfected with miR-30d-5p mimics or its negative control. Representative images of the migratory or invading cells (left panel), magnification: 400×. Histogram of the numbers of migratory or invading cells (right panel, **P* < 0.05).
